# Refracture following vertebral fragility fracture when bone fragility is not recognized: summarizing findings from comparator arms of randomized clinical trials

**DOI:** 10.1007/s40618-023-02222-0

**Published:** 2023-11-03

**Authors:** G. Porcu, A. Biffi, R. Ronco, G. Adami, R. Alvaro, R. Bogini, A. P. Caputi, B. Frediani, D. Gatti, S. Gonnelli, G. Iolascon, A. Lenzi, S. Leone, R. Michieli, S. Migliaccio, T. Nicoletti, M. Paoletta, A. Pennini, E. Piccirilli, M. Rossini, U. Tarantino, L. Cianferotti, M. L. Brandi, G. Corrao

**Affiliations:** 1 Department of Statistics and Quantitative Methods, National Centre for Healthcare Research and Pharmacoepidemiology, University of Milano-Bicocca, Milan, Italy; 2 Unit of Biostatistics, Epidemiology, and Public Health, Department of Statistics and Quantitative Methods, University of Milano-Bicocca, Milan, Italy; 3 Unit of Biostatistics, Epidemiology and Public Health, Department of Cardiac, Thoracic, Vascular Sciences and Public Health, University of Padua, Padua, Italy; 4 Rheumatology Unit, University of Verona, Verona, Italy; 5 Department of Biomedicine and Prevention, University of Rome Tor Vergata, Rome, Italy; 6 Local Health Unit (USL) Umbria, Perugia, Italy; 7 Department of Pharmacology, School of Medicine, University of Messina, Messina, Italy; 8 Department of Medicine, Surgery and Neurosciences, Rheumatology Unit, University of Siena, Azienda Ospedaliero-Universitaria Senese, Siena, Italy; 9 Department of Medicine, Surgery, and Neuroscience, Policlinico Le Scotte, University of Siena, Siena, Italy; 10 Department of Medical and Surgical Specialties and Dentistry, University of Campania “Luigi Vanvitelli”, Naples, Italy; 11 Department of Experimental Medicine, Sapienza University of Rome, Viale del Policlinico, Rome, Italy; 12 AMICI Onlus, Associazione nazionale per le Malattie Infiammatorie Croniche dell’Intestino, Milan, Italy; 13 Italian Society of General Medicine and Primary Care (SIMG), Florence, Italy; 14 Department of Movement, Human and Health Sciences, Foro Italico University, Rome, Italy; 15 CnAMC, Coordinamento nazionale delle Associazioni dei Malati Cronici e rari di Cittadinanzattiva, Rome, Italy; 16 Department of Clinical Sciences and Translational Medicine, University of Rome “Tor Vergata”, Rome, Italy; 17 Department of Orthopedics and Traumatology, “Policlinico Tor Vergata” Foundation, Rome, Italy; 18 Italian Foundation for Research on Bone Diseases (FIRMO), Florence, Italy

**Keywords:** Vertebral fragility fracture, Subsequent fractures, Refracture, Systematic review

## Abstract

**Purpose:**

Since vertebral fragility fractures (VFFs) might increase the risk of subsequent fractures, we evaluated the incidence rate and the refracture risk of subsequent vertebral and non-vertebral fragility fractures (nVFFs) in untreated patients with a previous VFF.

**Methods:**

We systematically searched PubMed, Embase, and Cochrane Library up to February 2022 for randomized clinical trials (RCTs) that analyzed the occurrence of subsequent fractures in untreated patients with prior VFFs. Two authors independently extracted data and appraised the risk of bias in the selected studies. Primary outcomes were subsequent VFFs, while secondary outcomes were further nVFFs. The outcome of refracture within ≥ 2 years after the index fracture was measured as (i) rate, expressed per 100 person-years (PYs), and (ii) risk, expressed in percentage.

**Results:**

Forty RCTs met our inclusion criteria, ranging from medium to high quality. Among untreated patients with prior VFFs, the rate of subsequent VFFs and nVFFs was 12 [95% confidence interval (CI) 9–16] and 6 (95% CI 5–8%) per 100 PYs, respectively. The higher the number of previous VFFs, the higher the incidence. Moreover, the risk of VFFs and nVFFs increased within 2 (16.6% and 8%) and 4 years (35.1% and 17.4%) based on the index VFF.

**Conclusion:**

The highest risk of subsequent VFFs or nVFFs was already detected within 2 years following the initial VFF. Thus, prompt interventions should be designed to improve the detection and treatment of VFFs, aiming to reduce the risk of future FFs and properly implement secondary preventive measures.

**Supplementary Information:**

The online version contains supplementary material available at 10.1007/s40618-023-02222-0.

## Introduction

Fragility fractures, resulting from a low-impact event such as a fall from a standing height, typically affect the elderly and individuals with poor bone quality [1]. The rising aging population in high-income countries is responsible for increasing rates of fragility fractures and their clinical and functional consequences [1, 2]. Indeed, older adults with an osteoporotic fracture are at higher risk of an imminent fracture, which is a subsequent event within 1–2 years after an index fracture [3, 4]. Therefore, secondary prevention should be adopted after an initial fracture to reduce the further risk of an imminent fracture [3]. In this regard, physicians should evaluate the fracture risk using an assessment tool [5] and are strongly encouraged to treat patients immediately after a sentinel event [6]. Thus, for patients with a high fracture risk, pharmacological agents ought to be promptly recommended as they could improve bone mineral density and reduce the incidence of subsequent fractures [4, 7].

Specifically, vertebral fragility fractures (VFFs), which are among the most common fragility fractures, (i) are the primary risk factor for the occurrence of further fractures affecting either vertebra or other sites [8–11], (ii) are associated with an increased risk of morbidity and mortality, and (iii) represent a significant economic burden on healthcare systems [12–16]. Although at least one in five persons aged > 50 years has ≥ 1 vertebral fracture [9, 16], detection of VFFs may be uneasy due to ambiguous terminology and the lack of diagnostic standards [2, 11, 14, 15, 17, 18]. Only a third of VFFs come to medical attention [16, 19], which might lead to inadequate patient care [18].

The current meta-analysis systematically reviewed randomized clinical trials (RCTs) investigating the efficacy of drugs for secondary prevention of refracture among patients who experienced vertebral fractures. We summarized data of patients who were randomly assigned to the control arm (i.e., who did not receive drugs for bone fragility care except calcium and vitamin D supplements), which are known to be ineffective for preventing fractures in community-dwelling adults [20]. Thus, we considered patients who received placebo as a proxy of patients with unrecognized fragility fractures. The aim of this systematic review and meta-analysis was to measure the implications of vertebral fractures on the risk of a new fracture in patients with unidentified frailty.

## Methods

### Search strategy and selection criteria

We followed the Preferred Reporting Items for Systematic Reviews and Meta-Analyses (PRISMA) checklist [21] for conducting and reporting this study. We did a systematic search of the Embase, PubMed (Medline), and Cochrane Library databases to cover primary studies, as well as systematic reviews published up to February 2022. A hand-checking search on clinicaltrial.gov was performed to detect additional eligible studies. The search strategy included keywords and/or corresponding MeSH terms related to “vertebral fragility fracture” and “subsequent fragility fractures”. Further details and search terms are listed in Supplemental Material.

Studies were eligible if they (i) were RTCs, (ii) reported data on refracture following a radiographically detected index fracture (or morphometric fracture) among (iii) patients with a VFF who, being randomly assigned to the comparator arm, did not have drug treatment for bone fragility. Vertebral and non-vertebral refracture occurring at the time point following the index fracture were considered primary and secondary outcomes, respectively. Studies were excluded if they (i) were not published in the English language, (ii) did not report original findings (i.e., letters and case reports), (iii) did not involve patients with at least one VFF at baseline, or (iv) did not evaluate the refracture risk. When data were published more than once, the most recent and complete paper was selected. Besides, if multiple articles were published on the same trial, all articles reporting different follow-up periods or different refracture sites were included.

Two independent authors (GP and AB) screened titles and abstracts according to the search strategy and then assessed the full text of all potentially relevant studies. Discrepancies between readers were resolved by conference. From each included RCT, the following information was extracted: (i) first author, year, and country of publication; (ii) type and characteristics of the target population; (iii) type of refracture; (iv) follow-up period.

### Study quality

The quality of each RCT was evaluated using the Cochrane risk of bias (RoB) tool for RCTs [22]. The following domains of the Cochrane RoB tool were appraised: selection bias (random sequence generation and allocation concealment), performance bias (blinding of participants and personnel), detection bias (blinding of outcome assessment), attrition bias (incomplete outcome data), reporting bias (selective reporting), and other bias (such as funding bias). Each domain was classified as “high” or “low” RoB. The latter was considered “unclear” if the publication did not provide sufficient information. The overall quality of each included study was judged as high, medium, or low if no high (and fewer than three unclear), at most one high (or more than three unclear), or more than one high RoB was found, respectively.

### Statistical analysis

Only patients belonging to the comparator arm for whom a radiograph exam was performed to clinically recognize the index fracture (i.e., fractured patients at risk of developing further fractures) were considered in the current meta-analysis.

The refracture outcome was measured through both rate and risk. The refracture rate was calculated as the number of patients assigned to the comparator arm who experienced a subsequent bone fracture over the person-years (PYs) from them accumulated. The refracture rate was expressed as per 100 PYs of follow-up and was presented with 95% confidence intervals (CIs). Unless directly reported in the original publication, PYs were derived as years accumulated during follow-up by patients at risk of developing the outcome (by right censuring observations at outcome occurrence or lost to follow-up when feasible). The refracture risk was calculated as the number of patients assigned to the comparator arm who experienced the outcome within two, three, and four years over the number of patients randomized to the comparator arm and expressed as a percentage with corresponding 95% CIs.

Estimates were summarized if at least three studies reported the estimate of interest. In the case of < 3 studies per category, data were aggregated into larger classes.

Subgroup analyses were planned for (i) the baseline number of VFFs and (ii) specific sites of no vertebral fracture during follow-up.

Heterogeneity between study-specific estimates was tested using Chi-square statistics [23] and measured with the I^2^ index (heterogeneity measure across studies) [24]. Studies were combined to obtain a summary estimate using the DerSimonian random-effects model [25]. Potential publication bias was visually and statistically identified through funnel plots and Egger’s test [26]. Furthermore, influence analysis was performed to assess the impact of a single study on the overall pooled estimates by omitting one study at a time.

All tests were considered statistically significant for *p*-values < 0.05. The analyses and the correspondent graphical visualization of forest plots were performed using RevMan V.5.4 (Nordic Cochrane Center, Copenhagen, Denmark) and R Statistical Software (v4.1.2; R Core Team 2021).

## Results

**Fig. 1:**
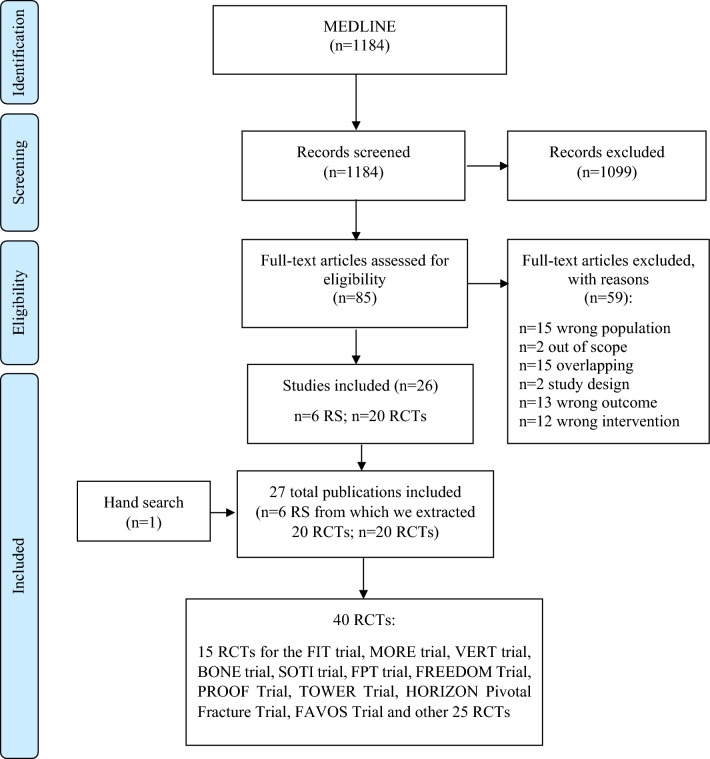
Flow chart

As shown in Fig. 1, a total of 1184 papers were initially extracted. Overall, after exclusion through title and abstract screening and further inclusion through papers referenced by systematic reviews [17, 27–31] and hand search on the topic [32], a total of 40 RCTs were included [32–72]. Summary characteristics of the 40 RCTs included in our meta-analysis are given in Table 1. Twenty-five and fifteen trials were classified into the categories of high or medium quality, respectively. Almost all RCTs had at least one unclear risk of bias, primarily regarding selection bias (random sequence generation or allocation concealment tools) or other bias. Moreover, 14 RCTs [34, 38, 40, 42, 44, 45, 48, 49, 55, 59–61, 63, 68] had a high risk of bias, mainly due to attrition (incomplete outcome data) (Supplemental Material, Figure S1–S2). Overall, 9,891 patients with at least one baseline VFF who did not receive drug treatment for correcting bone fragility except for supplements with calcium and/or vitamin D were considered to be summarized in our meta-analysis. These patients accumulated 22,990 PYs for the risk of refracture. Then, the majority of the included studies were focused on post-menopausal women.

**Table 1:** Characteristics of included randomized controlled trials

First author publication year, country	Study design	Population and exclusion criteria	Age range	Size M/F	Type of refracture	Estimate or proportion	Follow-up	Quality *
Black 1996, USA	FIT Trial	All women in the FIT trial were aged between 55 and 81 years at baseline, had been postmenopausal for at least 2 years, and had femoral neck BMD of 0·68 g/cm2 or less, about 2·1 SDs below peak bone mass based on the manufacturer’s normative data The authors excluded women with a single hospital admission for upper gastrointestinal bleeding or two or more documented ulcers within the preceding 5 years, dyspepsia requiring daily treatment, abnormal renal function (serum creatinine > 144 μmol/L), major medical problems that would be likely to preclude participation for 3 years, severe malabsorption syndrome, uncontrolled hypertension (blood pressure > 210 mm Hg systolic or > 105 mm Hg diastolic), myocardial infarction during the previous 6 months, unstable angina, or evidence of disturbed thyroid or parathyroid function. We also excluded women who had taken oestrogen or calcitonin within the preceding 6 months or bisphosphonates or sodium fluoride (> 1 mg daily for 2 weeks or longer) at any time	55–81	Total: 2027 0/2027 Placebo: 1005 0/1005	Vertebral and non-vertebral fractures	Vertebral fractures: 145/965 (15.0%) Non-vertebral fractures: 148/965 (15.3%) Hip fractures: 22/965 (2.3%) Wrist fractures: 41/965 (4.2%)	3 years	High
Ensrud 1997,	FIT trial	Postmenopausal women aged 55 to 81 years with low femoral neck bone mineral density (BMD) and existing vertebral fractures Pathological fractures, eg, those caused by malignancy, those caused by excessive trauma, and those involving the face and skull were excluded	55–81	Total: 2027 0/2027 Placebo: 1005 0/1005	Vertebral and any clinical fractures	Vertebral fractures: 1 prevalent VF: 59/965 (6.1%) ≥ 2 prevalent VF: 86/965 (9.2%);	3 years	High
Delmas 2003, 25 countries, mainly at sites in the United States and Europe	MORE Trial	Women with osteoporosis, as defined by low bone mineral density (femoral neck or lumbar spine BMD T-score ≤ -2.5) and/or radiographically apparent vertebral fractures, who were at least 2 years postmenopausal. In the present analyses, the relationship between osteoporosis risk factors at baseline, such as fracture severity grade, and the risk of new vertebral and non-vertebral fractures at 3 years, was evaluated in women randomized to the placebo group	≤ 81 years	Placebo:2576 0/2576	Vertebral and non-vertebral fractures	Vertebral fractures: Prevalent VF: 170/835 (20.4%) Non-vertebral fractures: Prevalent VF: 107/938 (11.4%) No Prevalent VF: 131/1627 (8.1%)	3 years	High
Sontag 2010, 180 sites in 25 countries	MORE Trial	Patients were enrolled into two sub-studies. One sub-study included patients whose femoral neck or lumbar spine BMD T-score was less than or equal to -2.5. The other sub-study included patients with low BMD and one or more moderate or severe vertebral fractures or two or more mild vertebral fractures, or who had at least two moderate fractures regardless of their BMD	Average: 67	Total: 5114 0/5114 Placebo: 2565 0/2565	Vertebral fractures	Vertebral fractures: Prevalent VF: 204/833 (24.5%) No Prevalent VF: 84/1459 (5.8%)	4 years	High
Harris 1993, USA	A 2-year, double-blind, multicenter study;	Enrollment was limited to white and Asian women with osteoporosis manifested as at least one but not more than 4 vertebral fractures. All were at least 12 months postmenopausal and generally healthy Women previously treated with sodium fluoride or any bisphosphonate were excluded. Other exclusion criteria were age greater than 75 years, weight below 40 kg or over 80 kg, and medical conditions that might contribute to accelerated bone loss or confound study participation	Postmenopausal ≤ 75 years	Total: 423 0/423 Placebo: 105 0/105	Vertebral fractures	Vertebral fractures: 20/92 (21.7%) Hip fractures: 2/105 (1.9%) Wrist fractures: 1/105 (0.9%)	3 years	High
McClung 2001, 183 study Centers in North America, Europe, New Zealand, and Australia	A clinical trial	Two groups of ambulatory postmenopausal women in two identical protocols at 183 study centers in North America, Europe, New Zealand, and Australia were enrolled. One group consisted of women 70 to 79 years old who had osteoporosis, indicated by either a bone mineral density at the femoral neck (T score) that was more than 4 SD below the mean peak value in young adults (-4) or a femoral-neck T score lower than -3 plus at least one risk factor for hip fracture. The other group consisted of women 80 years of age or older who had at least one non-skeletal risk factor for hip fracture, a femoral neck T score lower than -4, or a femoral-neck T score lower than -3 plus a hip-axis length of 11.1 cm or greater The exclusion criteria were any major medical illness, a recent history of cancer, another metabolic bone disease within the previous year, important abnormalities in the results of routine laboratory tests, recent use of drugs known to affect bone, allergy to any bisphosphonate, a history of bilateral hip fractures, and any physical or mental condition that would preclude participation in a clinical trial. There were no specific criteria for exclusion on the basis of previous or ongoing upper gastrointestinal tract disorders or concomitant use of nonsteroidal anti-inflammatory drugs, aspirin, proton-pump inhibitors, or antacids	≥ 70	Total: 9331 0/9331 Placebo: 3134 0/3134	Non-vertebral fractures	Non-vertebral fractures: 154/956 (16.1%) Hip fractures in women 70 to 79 years old with osteoporosis: Prevalent VF: 25/575 (4.3%) No Prevalent VF: 12/875 (1.4%)	3 years	High
Chesnut 2004 3 centers in Europe and North America	BONE trial		55–80	2946 0/2946	Vertebral and non-vertebral fractures	Vertebral fractures: 73/975 (7.5%)	3 years	High
Felsenberg 2005	BONE trial	The BONE study enrolled a total of 2946 patients aged 55–80 years, ≥ 5 years postmenopause, with 1–4 prevalent vertebral fractures (T4–L4), and with a BMD T score of -2.0 to -5.0 in ≥ 1 vertebra (L1–L4)	55–80	Placebo 982 0/982	Severe or moderate vertebral fractures	Vertebral fractures: 1 year: 24/975 (2.5%)	3 years	High
Meunier 2004, 11 European countries and Australia	SOTI Trial	Women were eligible for the study if they were at least 50 years old, had been postmenopausal for at least five years, had had at least one fracture confirmed by spinal radiography (after minimal trauma), and had a lumbar-spine bone mineral density of 0.840 g per square centimeter or less Women were ineligible if they had severe diseases or conditions that could interfere with bone metabolism or if they used antiosteoporotic treatments (fluoride salts and bisphosphonates taken for more than 14 days within the previous 12 months, or estrogen, calcitonin, or calcitriol taken for more than 1 month in the previous 6 months)	≥ 50	Total: 1442 0/1442 Placebo: 723 0/723	Vertebral and non-vertebral fractures	Vertebral fractures: 1 year: 88/723 (12.2%) 3 years: 237/723 (32.8%) Non-vertebral fractures: 122/723 (16.9%)	3 years	Medium
Meunier 2009	SOTI Trial	Post-menopausal (≥ 5 years) women were ambulatory Caucasian, ≥ 50 years of age with at least one prevalent osteoporotic vertebral fracture. Mean lumbar BMD had to be ≤ 0.840 g/cm2 Exclusion criteria were mainly concomitant pathologies or treatment potentially interfering with bone metabolism	≥ 50	Total: 1649 0/1649 Placebo: 726 0/726	Vertebral fractures	Vertebral fractures: 228/626 (36.4%)	4 years	Medium
Neer 2001 17 countries	Fracture Prevention Trial	Women were eligible for enrolment if they were ambulatory, if a period of at least five years had elapsed since menopause, and if they had at least one moderate or two mild atraumatic vertebral fractures on radiographs of the thoracic and lumbar spine, and an ambulatory status. For women with fewer than two moderate fractures, an additional criterion for enrolment was a value for bone mineral density of the hip or lumbar spine that was at least 1 SD below the mean value in normal premenopausal white women (age range, 20 to 35 years) Women with illnesses that affect bone or calcium metabolism, urolithiasis within the preceding 5 years, impaired hepatic function, a serum creatinine concentration exceeding 2 mg per deciliter (177 μmol per liter), or alcohol or drug abuse, as well as women who had taken drugs that alter bone metabolism within the previous 2 to 24 months (depending on the drug) were excluded	Mean: 70	Total: 1637 0/1637 Placebo: 544 0/544	Vertebral and non-vertebral fractures	Vertebral fractures: 64/448 (14.3%) Non-vertebral fractures: 30/544 (5.5%) Hip fractures: 4/544 (0.7%) Wrist fractures: 7/544 (1.3%)	21 ± 3 months	High
Prevrhal 2009, USA	Fracture Prevention Trial	Postmenopausal women with osteoporosis		Total: 1637 0/1637 Placebo: 544 0/544	Vertebral fractures	Vertebral fractures: Prevalent VF: 47/172 (27.3%) No prevalent VF: 4/123 (3.2%)	19 months	High
Wustrack 2012, 11 international centers in USA and Germany	HORIZON Pivotal Fracture Trial	All patients enrolled in this trial had osteoporosis as defined by a T-score of ≤ 2.5 measured at the hip or a T-score of ≤ 1.5 and a history of two mild or one moderate vertebral fracture. Patients did not taking any osteoporosis medications at time of randomization or met a defined washout criterion. The analysis was limited to those patients randomized to placebo	65–85	2677 0/2677	Vertebral fractures	Vertebral fractures: Prevalent VF: 190/1699 (11.2%) No prevalent VF: 38/978 (3.9%)	2 years	Medium
Chesnut 2000, USA and UK	PROOF Trial	White, Asian, or Hispanic women were eligible to participate if they were postmenopausal for at least 1 year and had one to five prevalent thoracic or lumbar vertebral compression fractures as evaluated at the study center, lumbar spine bone mineral density at least 2 SD below normal for normal women age 30 years, and no history of hip fracture Women with a history of diseases, conditions, or chronic usage of medications (eg, corticosteroids) that could affect bone metabolism or bone mass measurements were excluded, as were those who had been treated with calcitonin, estrogens, or fluorides within 3 months of study entry, continuous bisphosphonates for at least 3 months within 24 months, or cyclical bisphosphonates within 18 months	Placebo: Mean ± SD 68.2 6 7.7	Total: 1255 0/1255 Placebo: 311 0/311	Vertebral fractures	Vertebral fractures: 60/245 (24.5%)	3 years	High
Boonen 2011, USA	FREEDOM trial	Ambulatory postmenopausal women with a BMD T-score less than -2.5 at the lumbar spine or total hip but not less than -4.0 at either site were eligible to enroll in this study. Women with two or more vertebral deformities could be eligible, as long as there were no severe vertebral deformities and at most two moderate vertebral deformities For new vertebral fractures the higher-risk subgroups included women with the following: 1) two or more preexisting vertebral fractures of any degree of deformity, or one or more vertebral fracture of moderate or severe deformity, or both (prevalent vertebral fracture status); 2) a femoral neck BMD T-score of -2.5 or less; or 3) both multiple and/or moderate or severe vertebral deformities and a femoral neck BMD T-score of -2.5 or less. For hip fractures the higher-risk subgroups included women: 1) 75 yr old or older; 2) with a femoral neck BMD T-score of 2.5 or less; or 3) 75 yr old or older and with a femoral neck BMD T-score of -2.5 or less Women who did not have the risk factor(s) specified were included in the lower-risk subgroups	Mean (SD): 72.3 (5.2)	Total: 7808 0/7808 Placebo: 3580 0/3580	Vertebral and non-vertebral fractures	Vertebral fractures: Prevalent VF: 57/343 (16.6%) No prevalent VF: 202/3237 (6.2%); Hip fractures among women equal or older than 75 years: Prevalent VF: 26/1236 (2.1%); No prevalent VF: 17/2670 (0.6%);	3 years	High
Aloia 1988, USA	Double blind, randomized parallel trial	Women with postmenopausal osteoporosis between the ages of 50 and 80 years were recruited to participate in the study by media releases and letters to physicians. Osteoporosis was diagnosed by the presence of at least one non-traumatic vertebral compression fracture The women were otherwise healthy; specific exclusion factors included hepatic or renal disease, malignancy, malabsorption syndrome, parathyroid or thyroid disorders, inflammatory arthritis, alcoholism, overt vitamin D deficiency, a history of renal lithiasis, allergy to tetracycline, insulin-dependent diabetes, previous long-term hospitalization, and any other disorder known to affect bone metabolism. Patients were also excluded on the basis of intake df certain drugs, including glucocorticoids, anticonvulsants, estrogens, sodium fluoride, calcium supplements, and pharmacologic doses of vitamin D	50–80	Total: 27 0/27 Placebo: 15 0/15	Vertebral fractures	Vertebral fractures: 5/15 (33.3%)	2 years	High
Ott 1989, USA	Double-blind, randomized clinical trial	Eighty-six women recruited by media advertisements were enrolled. All were postmenopausal, between ages 50 and 80. Lateral radiographs of the spine were examined, and women were included if they had at least two compression fractures (> 15% reduction in anterior height) without history of serious trauma. All participants were white, were taking no medications for treatment of osteoporosis (except calcium supplements in some cases), and were ambulatory. All had normal results from tests for serum electrolytes, calcium, phosphate, alkaline phosphatase, liver function, creatinine, thyroid function, protein electrophoresis, urinalysis, hematocrit, and leukocyte count. Women with a history of corticosteroid use, malnutrition, sarcoidosis, liver disease, rheumatoid arthritis, nephrolithiasis, renal disease, or recent malignancy were excluded	50–80	Total: 86 0/86	Vertebral and non-vertebral fractures	Vertebral fractures: 15/86 (17.4%) Hip fractures: 0/86 (0.0%)	2 years	Medium
Gallagher 1990, USA	Two-year, double-blind, randomized clinical trial	Women with postmenopausal osteoporosis between 50 and 78 years of age were asked to participate in this study. Patients were referred directly to the bone clinic; advertisements were not used. The major criteria for inclusion in the study were that patients be postmenopausal and have one or more non-traumatic vertebral fractures Exclusion criteria included significant chronic disease such as renal failure, malignancy, gastrointestinal abnormalities, hyperparathyroidism or hypoparathyroidism, hyperthyroidism or hypothyroidism, acromegaly, the Cushing syndrome, or arthritis; evidence of overt vitamin D deficiency; or a history of renal calculi, diabetes, or alcoholism. Patients who had previously been immobilized for a prolonged period were also excluded as were patients who had taken corticosteroids for longer than 3 months; who had taken anticonvulsants, estrogen, calcium supplements, or vitamin D in the last 6 months; or sodium fluoride within the last year	50–78	50 0/50	Vertebral fracture	Vertebral fractures: 17/50 (34%)	2 years	Medium
Riggs 1990, USA	A prospective clinical trial	The 202 patients with Type I (postmenopausal) osteoporosis who were enrolled in this study were fully ambulatory, postmenopausal white women 50 to 75 years of age who had documented osteoporosis but no evidence of an associated disease or a history of use of any drug known to cause osteoporosis. The criteria for osteoporosis were diffuse osteopenia on spinal roentgenography, the presence of one or more vertebral fractures, defined as a decrease in vertebral height of more than 20 percent, and a bone-mineral-density value for the lumbar spine below the normal range for postmenopausal women	50–75	Total: 202 0/202 Placebo: 101 0/101	Non-vertebral fractures	Non-Vertebral fractures: 24/101 (23.8%) Hip fractures: 4/101 (4.0%) Wrist fractures: 4/101 (4.0%)	4 years	High
Storm 1990, Denmark	Double blind placebo-controlled study	Sixty-six postmenopausal women with osteoporosis (mean age, 68.3 years; range, 56 to 75) were enrolled in the randomized, double-blind, placebo-controlled study between October 1983 and April 1986. Inclusion in this study was based on evidence of osteoporosis, as determined by the presence of at least one but no more than four atraumatic vertebral crush fractures and radiographically confirmed demineralization of vertebrae. Patients were excluded if they had secondary causes of osteoporosis, such as hyperparathyroidism, Paget’s disease of bone, or renal osteodystrophy; impairment of renal, cardiac, or thyroid function; or a history of therapy with corticosteroids, estrogens, calcitonin, calcium, or vitamin D for three months or longer during the six months preceding study entry, or any such treatment during the two months preceding study entry. Patients were also excluded if they had received fluoride or disphosphonate therapy for any disease	56–75	Total: 66 0/66 Placebo: 33 0/33	Non-vertebral fractures	Non-vertebral fractures: 6/33 (18.2%) Hip fractures: 2/33 (6.1%)	150 weeks	Medium
Watts 1990, USA	A prospective double-blind placebo controlled trial	The patients were recruited by media announcements and letters to physicians. Enrollment was limited to white and Asian women with osteoporosis (defined as at least one but not more than four vertebral compression fractures plus radiographic evidence of vertebral osteopenia) who had been postmenopausal for at least 12 months and were generally healthy. Women who had been treated with estrogens, glucocorticoids, androgens, anabolic steroids, phosphate, or pharmacological doses of calcium (more than 1.0 g daily) or vitamin D (more than 1000 IU daily) during the preceding two months were excluded. Other exclusion criteria were an age of more than 75 years, weight below 40 kg or above 80 kg, secondary osteoporosis, and medical conditions that might confound study participation (i.e., active rheumatoid arthritis, active gastrointestinal or liver disease, chronic alcoholism, or renal impairment as evidence by a serum creatinine level of more than 210 µmol per liter)	≤ 75	Total: 429 0/429 Placebo: 104 0/104	Vertebral and non-vertebral fractures	Vertebral fractures: 10/91 (11.0%) Non-vertebral fractures: 16/104 (15.4%);	2 years	High
Kleerekoper 1991, USA	A prospective, randomized, double-blind, placebo-controlled clinical trial	The trial was restricted to white women aged 45 to 75 years at entry into the trial who were at least one year post-menopause. All had one or more vertebral compression fractures or two or more non-contiguous vertebral wedge deformities readily apparent on lateral spine radiographs and gave a history of none or trivial trauma at the time of fracture. Patients who had previously received therapy with sodium fluoride for osteoporosis were excluded from the trial as were patients who were on estrogen therapy for osteoporosis	45–75	Total: 84 0/84 Placebo: 38 0/38	Vertebral and non-vertebral fractures	Vertebral fractures: 22/33 (66.7%), Non-vertebral fractures: 7/38 (18.4%)	Median: 30 months	High
Lufkin 1992, USA	Double-blind, randomized, placebo-controlled clinical trial	The 75 women with established type I (postmenopausal) osteoporosis who were enrolled in our study were fully ambulatory, postmenopausal, white women 47 to 75 years of age who had documented osteoporosis but no evidence of an associated disease or a history of use of any drug known to cause osteoporosis or to affect calcium levels. The criteria for diagnosis of osteoporosis were a bone mineral density at the lumbar spine and proximal femur below the 10th percentile of normal premenopausal women and one or more vertebral fractures defined as a decrease in vertebral height of more than 15%	47–75	Total: 75 0/75 Placebo: 39 0/39	Vertebral fractures	Vertebral fractures 12/34 (35.3%)	1 year	Medium
Tilyard 1992, New Zealand	A prospective, multicentre, single-bling clinical trial	Ambulatory postmenopausal women 50 to 79 years old were enrolled. All had osteoporosis but not any disease associated with osteoporosis or other major medical problems and no history of using any drug known to cause or ameliorate osteoporosis; specifically, none of the women were taking estrogen. The diagnosis of osteoporosis was based on the presence of one or more non-tramautic vertebral compression fractures as seen on a lateral spinal roentgenogram; a fracture as defined as a reduction in the height of the anterior border of a vertebral body by 20 percent or more, as compared with the height of the posterior border. The patients were recruited from among 805 white women who had been screened because they had a history of previous fractures of the wrist or hip, loss of height, dowager’s hump, or chronic back pain, diagnosed by 123 primary care physicians in 1986 and 1987	50–79	622 0/622	Vertebral fractures	Vertebral fractures: 56/622 (9.0%)	3 years	Medium
Pak 1995, USA	Placebo-controlled randomized trial	110 women with postmenopausal osteoporosis were recruited into the trial. All had radiologic evidence of osteopenia and osteoporosis; one or more vertebral fractures believed to be non-traumatic; and no secondary cause of bone loss	Placebo: Mean ± SD 68.7 ± 8.9	110 0/110	Vertebral fractures	Vertebral fractures: 22/56 (39.3%)	Mean: 3 years	Medium
Recker 1996, USA	A prospective randomized, double-blind, placebo-controlled trial	The subjects were healthy white women volunteers of European ancestry, aged 73.5 ± 7.1 years, who were ambulatory and living independently. Enrollment was limited to healthy postmenopausal women over age 60 whose usual calcium intakes were estimated to be < 1 g/day. There was no upper age limit. The authors designed the study to evaluate spine fracture incidence and forearm bone mass changes in two groups; those with prevalent spine fractures (PF) and those without prevalent fractures (NPF) on entry. Subjects with other diagnoses or with treatments known to affect the skeleton were excluded	≥ 60	Total: 197 0/197 Placebo: 100 0/100	Vertebral fractures	Vertebral fractures Prevalent VF: 21/41 (51.2%); No prevalent VF: 13/61 (21.3%)	Mean (SD): 4.3 ± 1.1	High
Clemmesen 1997, Copenhagen County, Denmark, and Lie`ge, Belgium	A two center, double-masked, placebo-controlled, randomized trial	The study group comprised 132 otherwise healthy postmenopausal women, 53–81 years of age (mean age 68 years) and at least 1 year past the menopause, with established postmenopausal osteoporosis defined as at least one, but no more than four vertebral fractures, and at least three intact lumbar vertebrae	53–81	Total: 132 0/132 Placebo: 44 0/44	Vertebral and non-vertebral fractures	Vertebral fractures: 20/44 (45.4%) Non-vertebral fractures: 4/44 (9.1%)	1 year	High
Montessori 1997, Netherlands	An open, randomized, controlled, prospective trial	Between February 1991 and February 1992, 80 white, postmenopausal women with low bone mass were enrolled. To be eligible for the study, patients had to be less than 75 years old, ambulant and active, and postmenopausal (naturally or by bilateral oophorectomy) for at least 1 year, with a BMD of the lumbar spine > 1 SD below that of age-matched controls (Z-score < -1 SD) Systemic treatment with oestrogens, androgens, vitamin D, calcium in pharmacological doses (> 1 g/day), calcitonin or (other) bisphosphonates in the preceding year was not allowed. Patients suffering from secondary osteoporosis or other forms of metabolic bone disease, active gastrointestinal or liver disease, renal disease (serum creatinine > 115 umol/1), active cancer within the last 3 years, or alcoholism, were excluded also	≤ 75	Total: 80 0/80 Placebo: 39 0/39	Vertebral fractures	Vertebral fractures: Prevalent VF: 3/17 (17.6%) No Prevalent VF: 0/22 (0%)	2 years	Medium
Lufkin 1998, USA	A 1-year prospective, randomized, double-blind trial	One hundred and forty-three women with postmenopausal osteoporosis were enrolled in the clinical trial. Subjects were eligible if they were in good health except for osteoporosis, free of any serious acute or chronic medical condition that might affect bone or calcium metabolism, fully ambulatory, between the ages of 45 and 75 years, and postmenopausal (no menses for 5 years or levels of serum estradiol, < 73 pmol/l and serum follicle-stimulating hormone [FSH] > 30 IU/l). The criteria for the diagnosis of osteoporosis were a bone mineral density (BMD) value for either the lumbar spine or proximal femur of ≤ 10th percentile for normal premenopausal females and one or more non-traumatic vertebral fractures, defined as a decrease in vertical height of ≥ 15% compared with adjacent vertebrae Specific exclusion criteria included patients with a history of deep venous thrombosis, thromboembolic disorders, or cerebral vascular accident, also patients with a history of cancer within the previous 5 years, except for superficial skin cancer	45–75	Total: 143 0/143 Placebo: 48 0/48	Vertebral fractures	Vertebral fractures: 18/48 (37.5%)	1 year	High
Meunier 1998, UK	FAVOS (Fluoride And Vertebral Osteoporosis Study	The 354 patients enrolled in 134 clinical centers were ambulatory postmenopausal white women (mean age 65.7 + 5.7 years; range 47–76 years) who had postmenopausal osteoporosis. The inclusion criteria were the presence of one to four vertebral fractures defined as a decrease in vertebral height of more than 25%, and a low BMD value for the lumbar spine in patients having only one vertebral fracture The exclusion criteria were a history of hip fracture, evidence of an associated disease (renal failure with a serum creatinine 413 mmol/dl, current malignant disease, thyrotoxicosis, osteomalacia, primary hyperparathyroidism, severe scoliosis) and use of any drug known to influence bone remodeling (corticosteroids, thyroxine). In addition, none of the women had received fluoride salts during the 5 years preceding recruitment. In addition, in order to not recruit patients with an extremely low lumbar BMD, a T-score below -5 SD was considered as an exclusion criterion	47–76	Total: 354 0/354 Placebo: 146 0/146	Non-vertebral fractures	Non-vertebral fractures: 17/146 (11.6%) Hip fractures: 2/146 (1.4%) Wrist fractures: 4/146 (2.7%)	3 years	High
Wimalawansa 1998, England	A prospective randomized study	After screening 350 new patients, 72 postmenopausal Caucasian women (mean age 64.960.5 years, range 58 to 72; median number of years since menopause 15) with established osteoporosis were enrolled. Inclusion criteria were evidence of osteoporosis as determined by at least 1 (but not more than 4) radiographically demonstrable atraumatic thoracic vertebral crush fractures, and spine BMD 2.0 standard deviations below the reference range established with normal healthy women aged 35 years, using dual energy X-ray absorptiometry bone density scanning (DXA) Women who had surgical menopause (ie, oophorectomy), secondary osteoporosis, or other medical conditions that can affect the skeleton, or were taking medications that affect calcium metabolism within the previous 3 years were excluded from this study (1,2,34). Patients treated with HRT, anabolic steroids, glucocorticoids, calcitonin, fluoride, or bisphosphonates at any time since menopause were also excluded	58–72	Total: 72 0/72 Placebo: 18 0/18	Non-vertebral fractures	Non-vertebral fractures: 1/18 (5.5%)	4 years	Medium
Ringe 1999, Germany	A randomized prospective study	Postmenopausal women with established osteoporosis were included The patients were recruited from the outpatients who fulfilled the criteria of being postmenopausal women not older than 75 years with primary generalized osteoporosis (BMD at L2–4: T-score < -2.5) presenting at least one osteoporotic vertebral fracture. Patients with a disorder or a medication that might significantly influence bone metabolism were excluded from the study	≤ 75	Total 145 0/145 Placebo: 45 0/45	Vertebral and non-vertebral fractures	Vertebral fractures: 30/45 (66.7%); Non vertebral fractures 22/45 (48.9%)	3 years	High
McCloskey 2004 UK	A double-blind, placebo-controlled study	The study included women with postmenopausal or secondary osteoporosis recruited from women referred to each study center for investigation and treatment of probable osteoporosis. All women had densitometrically proven vertebral osteoporosis (spine T-score ≤ -2.5, using the reference ranges provided by the manufacturers) and/or had at least one prevalent vertebral fracture at entry		Placebo 301 0/301	Vertebral fractures	Vertebral fractures: Prevalent VF: 49/149 (32.9%) No prevalent VF: 12/152 (7.9%)	3 years	High
Brumsen 2002, The Netherlands	A 3-year randomized, double-blind, placebo controlled, clinical trial	Patients were recruited from those referred to the participating centers for investigation and treatment of osteoporosis Included were women younger than 75 years and at least 5 years postmenopausal and men aged between 40 and 75 years with at least one atraumatic radiologically documented vertebral fracture. Patients had to have a life expectancy of at least 5 years Excluded were patients with abnormal liver function; serum creatinine > 140 µM; history of any malignant disease; disorders of calcium and bone metabolism other than osteoporosis; endocrine disorders; treatment with antiepileptics or glucocorticoids at a dose of > 7 mg/day of prednisolone or equivalent for 1 week or longer in the year preceding the trial; or prior treatment with bone-acting drugs such as sodium fluoride during the previous 9 months or any use for > 3 months, bisphosphonates in the previous 3 years, anabolic steroids or estrogens in the previous 6 months, or calcitonin in the last 3 months before entry into the study. Peptic ulcer or gastrointestinal diseases, other than malabsorption syndromes, were not considered exclusion criteria	≤ 75	Total: 101 23/78 Placebo: 50 11/39	Vertebral and non-vertebral fractures	Vertebral fractures 15/45 (33.3%); Hip fracture: 1/45 (2.2%) Wrist fracture: 2/45 (4.4%)	3 years	High
Gutteridge 2002, Australia	A Randomized Trial	Patients were postmenopausal Caucasian women under 80 years with at least one vertebral fracture Exclusion criteria included past hip fracture, renal impairment (plasma creatinine 4130 mmol/l), prior treatment with NaF (45 mg/day), past treatment with oral corticosteroids, anti-epileptics, or high-dose vitamin D (over 1000 IU/day for 41 month in the past year), and other medications or diseases associated with altered calcium metabolism	≤ 80	Total: 99 0/99 Placebo: 31 0/31	Vertebral and non-vertebral fractures	Vertebral fractures: 3/31 (9.7%) Non-vertebral fractures: 6/31 (19.3%) Wrist fractures: 2/31 (6.4%)	27 months	Medium
Greenspan 2007, 168 centers in 9 countries worldwide	18-month, randomized, double-blind, placebo-controlled, parallel-group study	The authors included postmenopausal women 45 to 54 years of age if bone mineral density (BMD) was 3.0 SDs or more (T-score ≤ -3.0) below the mean peak bone mass of young adult women at the lumbar spine, femoral neck, or total hip with no prevalent vertebral fracture or if BMD T-score was -2.5 and they had 1 to 4 vertebral fractures before enrollment. They also included postmenopausal women 55 years of age or older if BMD T-score was -2.5 and they had no vertebral fractures or if BMD T-score was -2.0 and they had 1 to 4 vertebral fractures The authors excluded women if baseline serum calcium level was greater than 2.66 mmol/L (> 10.7 mg/dL) or if urinary calcium– creatinine ratio was 1.0 or more. They included women with mild hypercalcemia (serum calcium, 2.55 to 2.66 mmol/L [10.2 to 10.7 mg/dL]) and mild hypercalciuria (24-h urine calcium ≥ 7.6 mmol [≥ 302 mg]) at baseline. We did not measure baseline levels of serum PTH and vitamin D. We excluded women if they had taken bisphosphonates for a total of more than 12 months or for more than 90 days in the 12 months before enrollment They allowed previous estrogen therapy if it had been discontinued for at least 4 weeks before the screening visit. Women who had received PTH (or a peptide fragment or analogue), PTH-related protein, fluoride, or strontium and those who had a history of metabolic bone disease (other than osteoporosis), nephrolithiasis, or clinically significant hepatic or renal disorders were also excluded, as well as women who were taking medications known to affect bone mineral metabolism	≥ 45	Total: 2532 0/2532 Placebo: 1246 0/1246	Vertebral fractures	Vertebral fractures: Prevalent VF: 21/235 (8.9%) No Prevalent VF: 21/1011 (2.1%)	18 months	Medium
Matsumoto 2009, Japan	A randomized, double-blind, pla cebo-controlled tria	We studied postmenopausal women aged 55 to 80 with one to five fragility fractures between the vertebrae T4 and L4 and BMD below 80% (T score − 1.7 at the lumbar spine) of the young adult mean (YAM) Subjects were excluded if they had disorders such as primary hyperparathyroidism, Cushing’s syndrome, premature menopause due to hypothalamic, pituitary or gonadal insufficiency, poorly controlled diabetes mellitus (HbA1c over 8.0%), or other causes of secondary osteoporosis, or if they had any radiographic finding that might affect the assessment of vertebral fractures and used hard or semihard corset in spine part. Subjects with peptic ulcer were excluded. Subjects were excluded if they had taken bisphosphonates at any time. Subjects were also excluded if they had taken glucocorticoids, calcitonin, vitamin K, active vitamin D compounds, or hormone replacement therapy within the previous 2 months, had serum calcium (Ca) levels above 10.6 mg/dL (2.7 mmol/L) or below 8.0 mg/dl (2.0 mmol/L), had serum creatinine levels above 1.5 mg/dL (133 μmol/L), or had clinically significant hepatic disorders	55–80	Total: 704 0/704 Placebo: 345 0/345	Vertebral and non-vertebral fractures	Vertebral fractures: 69/345 (20.0%); Non vertebral fractures: 12/345 (3.5%)	2 years	Medium
Hagino 2013, Japan	A post-hoc analysis of a randomized, double-blind, placebo-controlled study	The subjects in the original study were women aged 55–80 years old with 1–5 fragility fractures between the T4 and L4 vertebrae, and BMD < 80% of the young adult mean (YAM)	55–80	Total: 704 0/704 Placebo: 345 0/345	Vertebral fractures	Vertebral fractures: 1 prevalent VF: 45/345 (13%) 2 prevalent VF: 80/345 (23.2%) 3 prevalent VF: 142/345 (41.1%)	2 years	Medium
Fujita 2014, Japan	A randomized, double-blind trial	Men and postmenopausal women with primary osteoporosis with one to five vertebral fractures and capable of self-supported walking were eligible for enrollment Patients with secondary osteoporosis; hypersensitivity, such as bronchial asthma or rashes; hypercalcemia; probable pregnancy; primary hypo- or hyperparathyroidism; past history of drug allergy; severe complications, such as cardiac, renal, or liver dysfunction; mental disease; dementia; or severe deformity of the spine were excluded. Patients who had taken bisphosphonates within 48 weeks prior to entry in the study and those treated with calcitonin, active vitamin D derivatives, vitamin K, raloxifene, sex hormone replacement, or anabolic steroids within the previous 8 weeks were also excluded	Placebo: Mean ± SD 71.3 ± 6.9	Total: 293 15/278 Placebo: 143 9/134	Vertebral and non-vertebral fractures	Vertebral fractures: 18/143 (12.6%) Non-vertebral fractures: 8/143 (5.6%)	78 weeks	High
Nakano 2014, Japan	TOWER Trial	The TOWER trial subjects included men and postmenopausal women with primary osteoporosis between 65 and 91 years of age, who had one to five vertebral fractures, and low BMD (T- score < -1.67) at the lumbar spine (L2–L4), femoral neck, total hip, or distal radius	65–91	Total: 578 0/578 Placebo: 290 0/290	Vertebral fractures	Vertebral fractures: 37/281 (13.2%)	72 weeks	High

*High: less than 3 unclear risk and no high risk; Medium: more than 3 unclear risk or at most one high risk; Low: more than one high risk

**Fig. 2:**
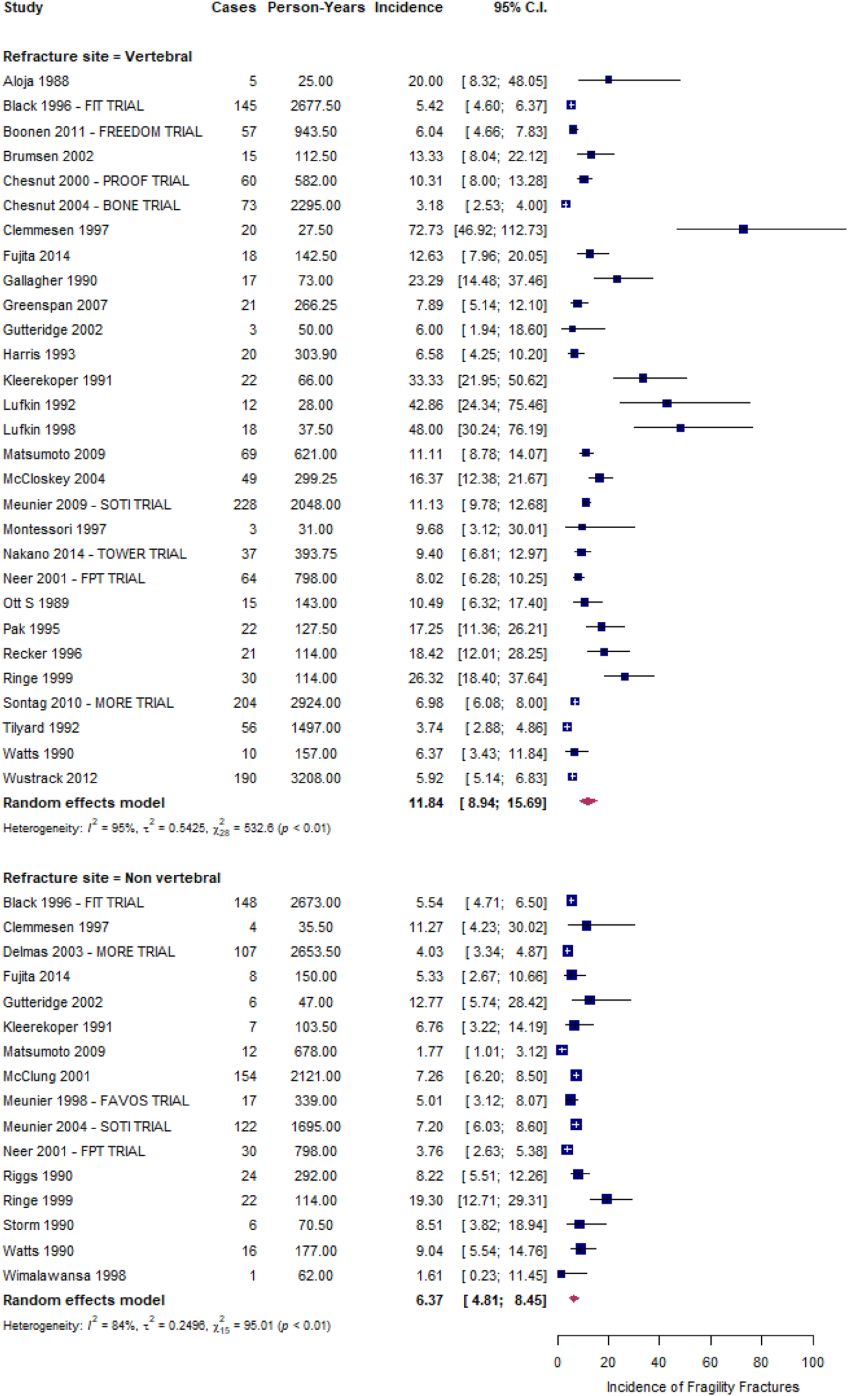
The incidence rate of new vertebral and non-VFFs, expressed as number of patients every 100 person-years

Figure 2 shows the forest plots of vertebral and non-vertebral refracture rates. Vertebral fracture rates ranged from 3 to 73 refractures per 100 PYs, being an overall estimate of 12 (95% CI 9–16) refractures per 100 PYs and having very high between-studies heterogeneity (I^2^ ≥ 90%) (Fig. 2, upper box). During the follow-up period, an increase in the rate of vertebral fractures was observed, specifically 4 (2–7) per 100 PY for those with 1 fracture at baseline and 13 (6–29) per 100 PY for those with more than 2 fractures at baseline (Supplementary Material, Figure S3). nVFFs rates ranged from 2 to 19 refractures per 100 PYs, being the overall estimate of 6 (95% CI: 5–8) refractures per 100 PYs and having high between-studies heterogeneity (I^2^ ≥ 75%) (Fig. 2, bottom box).

The summary fracture rate of (i) upper limbs was 1.3 (95% CI 1.0–1.7), 2.5 (0.7–9.4), and 0.8 (0.3–2.0) per 100 PYs for wrist, arm or forearm, and humerus, respectively (Supplemental Material, Figure S4); (ii) lower limbs was 0.9 (0.7–1.2), 0.4 (0.2–0.6), 0.4 (0.2–0.6), and 0.8 (0.4–1.7) per 100 PYs for hip, ankle, pelvis, foot or metatarsal, respectively (Supplemental Material, Figure S5); (iii) torso was 1.4 (0.5–4.1) (Supplemental Material, Figure S6); and (iv) other fractures was 2.6 (1.6–4.3) (Supplemental Material, Figure S7). The between-studies heterogeneity was reduced for upper and lower limb fractures (I^2^ < 75%).

Publication bias was detected for vertebral fractures (*p*-value = 0.0124, Supplemental Material, Figures S8–S9), although there was no evidence of the influence of any individual study (Supplemental Material, Figure S10) for both vertebral and non-vertebral refractures.

**Fig. 3:**
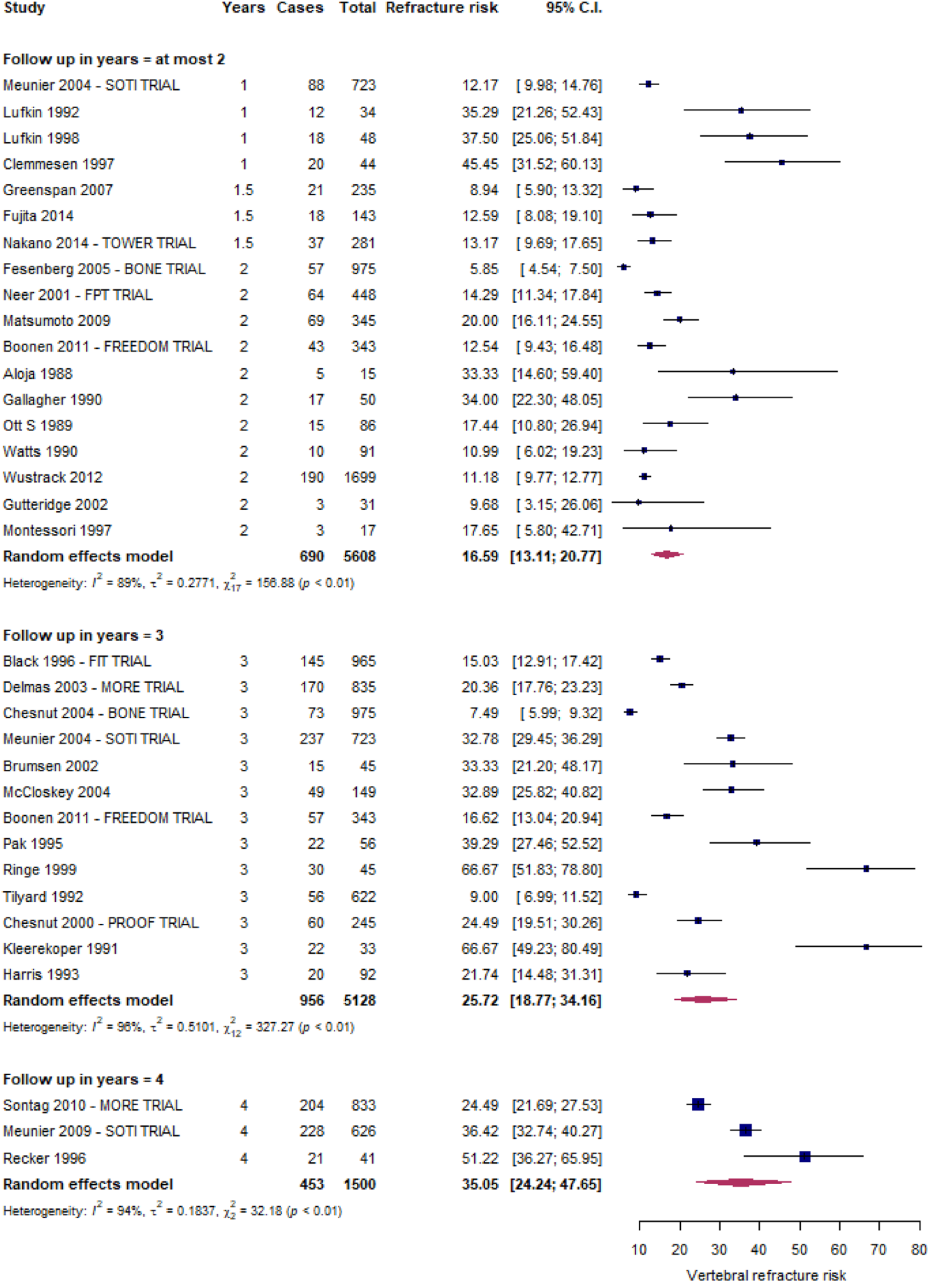
Risk of new VFFs

**Fig. 4:**
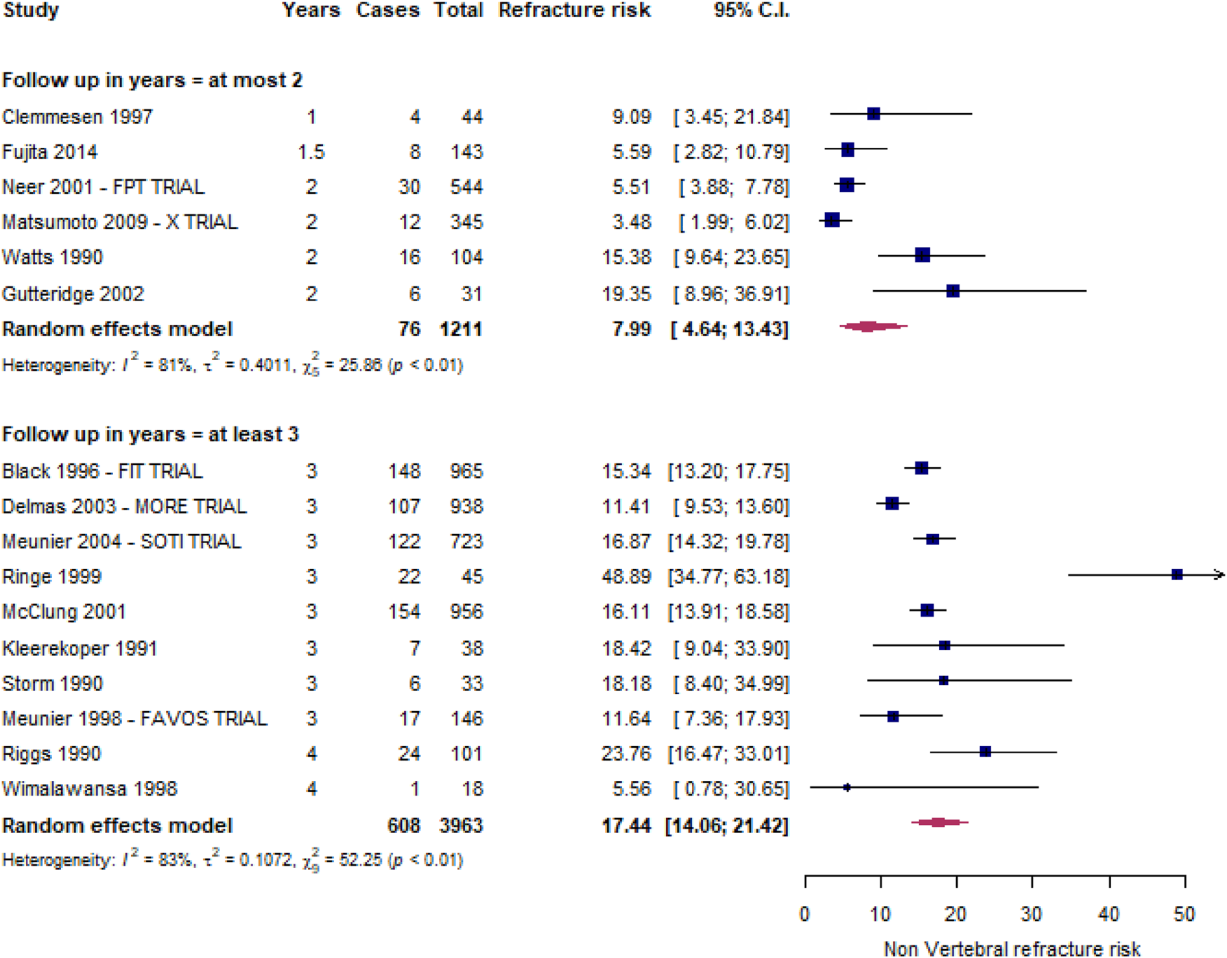
Risk of new nVFFs

Figures 3 and 4 depict the forest plots of vertebral and non-vertebral refracture risks, respectively. Within two, three, and four years from the index fracture, 16.6% (13.1–20.8%), 25.7% (18.8–34.1%), and 35.1% (24.4–47.7%) of patients, respectively, experienced at least a vertebral refracture, while the non-vertebral fracture risk was 8.0% (4.6–13.4%) within 2 years and 17.4% (14.1–21.4%) over 2 years.

## Discussion

In this meta-analysis of 40 original RCTs including almost 10,000 untreated patients affected by vertebral fracture at baseline, we found that, on average, a new vertebral and non-vertebral fracture occurred every year in 12 and 6 patients, respectively, per 100 patients who had previously experienced a vertebral fracture. Our meta-analysis found that 16.6% and 35.1% of patients experienced at least a vertebral refracture within two and four years from the index fracture, respectively, while non-vertebral fracture risk was 8.0% within 2 years and 17.4% over 2 years.

Our results are confirmed by the findings in the literature. Particularly, the UK clinical guideline for the prevention and treatment of osteoporosis revealed a doubled fracture risk related to the prior fracture, particularly for > 1 vertebral fracture [73]. Then, a summary of the literature reported a strong association, approximately 4 times greater, between prior and subsequent vertebral fractures than those without prior fractures, particularly within the next 2 years after the initial fracture [74]. The risk of further vertebral fractures appeared to increase with the number of prior vertebral fractures [74].

Early identification of VFFs might present a real opportunity to reduce the risk of a subsequent fracture [75]. However, there is considerable evidence that vertebral fractures might not be properly considered by clinicians and are under-reported by radiologists [14, 76], who might not use specific terminology and not alert the referring healthcare professionals. Thus, standardized radiographic acquisition and unambiguous radiological interpretation could contribute to reducing the further risk of VFFs [76].

Because the patients included in our meta-analysis received a drug therapy that should be considered ineffective for the treatment of fragility, all these findings strongly suggest that recognizing fragility as the cause or concomitant cause of the vertebral fracture should be considered a priority for the secondary prevention of fracture.

### Strengths and limitations

The findings of this study should be interpreted considering its limitations. First, the analysis was not patient-centered but instead used summary data; therefore, an accurate assessment might be lacking due to the nature of meta-analysis. Second, this systematic review selected RCTs that included patients who might have different characteristics compared to the general population. Third, our results were affected by high heterogeneity. Particularly, there are certain concerns as to whether findings from selected studies could be combined into one conclusion since primary findings were obtained from studies including heterogeneous populations, definitions of vertebral fractures and adopting different study designs. Fourth, among trials in which PYs were not reported, we assumed that censorships occurred in a mean of half of the entire follow-up period and could estimate below or above the incidence rate. However, this assumption can reasonably be considered valid in the case of large data and/or time intervals of limited amplitude. At last, an unclear risk of bias was found in nearly all the included studies, primarily regarding attrition bias, and a high risk of bias was detected in 12 RCTs, mainly due to attrition bias.

Despite these limitations, this study had certain strengths. The exhaustive search strategy provided an overview of RCTs on the subsequent fractures among untreated patients with prior VFFs. In addition, the internal validity of the selected studies was assessed using the RoB tool for RCTs.

## Conclusion

The average annual rate and short-long term risk of refracture among untreated patients with VFFs were estimated from this meta-analysis. Based upon the currently available evidence, further fractures are commonly observed in the following two years after the initial VFF. Early and accurate detection of VFFs should be conducted to reduce the risk of future fragility fractures and properly establish secondary prevention.

### Supplementary Information

Below is the link to the electronic supplementary material.

Supplementary file 1: (DOCX 899 KB)

## Data Availability

No additional data is available.

## Declarations

### Patient and public involvement statement

This research was done without patient involvement. Patients were not invited to comment on the study design and were not consulted to develop patient-relevant outcomes or interpret the results. Patients were not invited to contribute to the writing or editing of this document for readability or accuracy.
