# Supplementary material for: Refracture following vertebral fragility fracture when bone fragility is not recognized: summarizing findings from comparator arms of randomized clinical trials
Source: J Endocrinol Invest. 2023 Nov 3;47(4):795–818. doi: 10.1007/s40618-023-02222-0 (PMC10965723; doi:10.1007/s40618-023-02222-0)
Supplement: Supplementary file 1 — (DOCX 899 KB) [file 40618_2023_2222_MOESM1_ESM.docx]

**Supplemental Material**

**Refracture following vertebral fragility fracture when bone fragility is not recognized. Summarizing findings from comparator arms of randomized clinical trials**

Porcu G^1,2,3*^, Biffi A^1,2^, Ronco R^1,2^, Adami G^4^, Alvaro R^5^, Bogini R^6^, Caputi AP^7^, Frediani B^8^, Gatti D^4^, Gonnelli S^9^, Giovanni Iolascon^10^, Lenzi A^11^, Leone S^12^, Michieli R^13^, Migliaccio S^14^, Nicoletti T^15^, Paoletta M^10^, Pennini A^4^, Piccirilli E^16^, Rossini M^4^, Tarantino U^16^, Cianferotti L^17^, Brandi ML^17^, Corrao G^1,2^

^1^Department of Statistics and Quantitative Methods, National Centre for Healthcare Research and Pharmacoepidemiology, University of Milano-Bicocca, Milan, Italy

^2^Unit of Biostatistics, Epidemiology, and Public Health, Department of Statistics and Quantitative Methods, University of Milano-Bicocca, Milan, Italy

^3^Unit of Biostatistics, Epidemiology and Public Health, Department of Cardiac, Thoracic, Vascular

Sciences and Public Health, University of Padua, Padua, Italy

^4^Rheumatology Unit, University of Verona, Verona, Italy

^5^Department of Biomedicine and Prevention, University of Rome Tor Vergata, Rome, Italy

^6^Local Health Unit (USL) Umbria, Italy

^7^Department of Pharmacology, School of Medicine, University of Messina, Italy

^8^Department of Medicine, Surgery and Neurosciences, Rheumatology Unit, University of Siena, Azienda Ospedaliero-Universitaria Senese, Siena, Italy

^9^Department of Medicine, Surgery, and Neuroscience, Policlinico Le Scotte, University of Siena, Siena, Italy

^10^Department of Medical and Surgical Specialties and Dentistry, University of Campania “Luigi Vanvitelli,” Naples, Italy

^11^Department of Experimental Medicine, Sapienza University of Rome, Viale del Policlinico, Rome, Italy

^12^AMICI Onlus, Associazione nazionale per le Malattie Infiammatorie Croniche dell'Intestino, Milan, Italy

^13^Italian Society of General Medicine and Primary Care (SIMG), Florence, Italy

^14^Department of Movement, Human and Health Sciences, Foro Italico University, Rome, Italy

^15^CnAMC, Coordinamento nazionale delle Associazioni dei Malati Cronici e rari di Cittadinanzattiva, Italy

^16^Department of Clinical Sciences and Translational Medicine, University of Rome “Tor Vergata,” Rome, Italy; Department of Orthopedics and Traumatology, “Policlinico Tor Vergata” Foundation, Rome, Italy

^17^Italian Bone Disease Research Foundation (FIRMO), Florence, Italy

* Corresponding author e-mail address: [gloria.porcu@unimib.it](mailto:gloria.porcu@unimib.it)

**[Table S1.](#_Toc137030084)** [PRISMA checklist. 3](#_Toc137030084)

[**Table S2.** Search Strategy 3](#_Toc137030085)

[**Figure S1**. Quality evaluation of Randomized Controlled Trials 4](#_Toc137030086)

[**Figure S2.** Overall quality of Randomized Controlled Trials 5](#_Toc137030087)

[**Figure S3.** Incidence rate of vertebral fragility fractures (VFF) according to number of prior VFF, expressed as number of patients every 100 person-years 5](#_Toc137030088)

[**Figure S4.** Incidence rate of upper limbs fractures, expressed as number of patients every 100 person-years 6](#_Toc137030089)

[**Figure S5.** Incidence rate of lower limbs fractures, expressed as number of patients every 100 person-years 7](#_Toc137030090)

[**Figure S6.** Incidence rate of torso fractures, expressed as number of patients every 100 person-years 8](#_Toc137030091)

[**Figure S7.** Incidence rate of other fractures, expressed as number of patients every 100 person-years 8](#_Toc137030092)

[**Figure S8.** Begg’s funnel plot for publication bias in the studies assessing incidence rate of new vertebral fracture. 9](#_Toc137030093)

[**Figure S9.** Begg’s funnel plot for publication bias in the studies assessing incidence rate of new no vertebral fracture. 9](#_Toc137030094)

[**Figure S10.** Influence analysis investigating the pooled incidence rate of a) vertebral refracture b) no vertebral refracture by omitting one study at time among those contributing the estimates reported in Figure 2. 10](#_Toc137030095)

[Complete list of experts involved 11](#_Toc137030096)

# **Table S1.** PRISMA checklist.

| **Section and Topic** | **Item #** | **Checklist item** | **Location where item is reported** |
| --- | --- | --- | --- |
| **TITLE** | | |  |
| Title | 1 | Identify the report as a systematic review. | 1 |
| **ABSTRACT** | | |  |
| Abstract | 2 | See the PRISMA 2020 for Abstracts checklist. | 2 |
| **INTRODUCTION** | | |  |
| Rationale | 3 | Describe the rationale for the review in the context of existing knowledge. | 3 |
| Objectives | 4 | Provide an explicit statement of the objective(s) or question(s) the review addresses. | 3 |
| **METHODS** | | |  |
| Eligibility criteria | 5 | Specify the inclusion and exclusion criteria for the review and how studies were grouped for the syntheses. | 3 |
| Information sources | 6 | Specify all databases, registers, websites, organisations, reference lists and other sources searched or consulted to identify studies. Specify the date when each source was last searched or consulted. | 3-4 |
| Search strategy | 7 | Present the full search strategies for all databases, registers and websites, including any filters and limits used. | Table S2 |
| Selection process | 8 | Specify the methods used to decide whether a study met the inclusion criteria of the review, including how many reviewers screened each record and each report retrieved, whether they worked independently, and if applicable, details of automation tools used in the process. | 4 |
| Data collection process | 9 | Specify the methods used to collect data from reports, including how many reviewers collected data from each report, whether they worked independently, any processes for obtaining or confirming data from study investigators, and if applicable, details of automation tools used in the process. | 4 |
| Data items | 10a | List and define all outcomes for which data were sought. Specify whether all results that were compatible with each outcome domain in each study were sought (e.g. for all measures, time points, analyses), and if not, the methods used to decide which results to collect. | 3-4 |
|  | 10b | List and define all other variables for which data were sought (e.g. participant and intervention characteristics, funding sources). Describe any assumptions made about any missing or unclear information. | 3-4 |
| Study risk of bias assessment | 11 | Specify the methods used to assess risk of bias in the included studies, including details of the tool(s) used, how many reviewers assessed each study and whether they worked independently, and if applicable, details of automation tools used in the process. | 4 |
| Effect measures | 12 | Specify for each outcome the effect measure(s) (e.g. risk ratio, mean difference) used in the synthesis or presentation of results. | 4-5 |
| Synthesis methods | 13a | Describe the processes used to decide which studies were eligible for each synthesis (e.g. tabulating the study intervention characteristics and comparing against the planned groups for each synthesis (item #5)). | 5 |
|  | 13b | Describe any methods required to prepare the data for presentation or synthesis, such as handling of missing summary statistics, or data conversions. | 5 |
|  | 13c | Describe any methods used to tabulate or visually display results of individual studies and syntheses. | 5 |
|  | 13d | Describe any methods used to synthesize results and provide a rationale for the choice(s). If meta-analysis was performed, describe the model(s), method(s) to identify the presence and extent of statistical heterogeneity, and software package(s) used. | 5 |
|  | 13e | Describe any methods used to explore possible causes of heterogeneity among study results (e.g. subgroup analysis, meta-regression). | 5 |
|  | 13f | Describe any sensitivity analyses conducted to assess robustness of the synthesized results. | 5 |
| Reporting bias assessment | 14 | Describe any methods used to assess risk of bias due to missing results in a synthesis (arising from reporting biases). | 4 |
| Certainty assessment | 15 | Describe any methods used to assess certainty (or confidence) in the body of evidence for an outcome. | 5 |
| **RESULTS** | | |  |
| Study selection | 16a | Describe the results of the search and selection process, from the number of records identified in the search to the number of studies included in the review, ideally using a flow diagram. | 5 |
|  | 16b | Cite studies that might appear to meet the inclusion criteria, but which were excluded, and explain why they were excluded. | 5 - Figure 1 |
| Study characteristics | 17 | Cite each included study and present its characteristics. | 5 - Table 1 |
| Risk of bias in studies | 18 | Present assessments of risk of bias for each included study. | 5  Figures S1 |
| Results of individual studies | 19 | For all outcomes, present, for each study: (a) summary statistics for each group (where appropriate) and (b) an effect estimate and its precision (e.g. confidence/credible interval), ideally using structured tables or plots. | 5-6 – Figures 2-4, S3-S7 |
| Results of syntheses | 20a | For each synthesis, briefly summarise the characteristics and risk of bias among contributing studies. | 5  Figures S2 |
|  | 20b | Present results of all statistical syntheses conducted. If meta-analysis was done, present for each the summary estimate and its precision (e.g. confidence/credible interval) and measures of statistical heterogeneity. If comparing groups, describe the direction of the effect. | 5-6 – Figures 2-4, S3-S7 |
|  | 20c | Present results of all investigations of possible causes of heterogeneity among study results. | 5-6 |
|  | 20d | Present results of all sensitivity analyses conducted to assess the robustness of the synthesized results. | 5-6 –  Figures S3-S7 |
| Reporting biases | 21 | Present assessments of risk of bias due to missing results (arising from reporting biases) for each synthesis assessed. | Figures S1-S2 |
| Certainty of evidence | 22 | Present assessments of certainty (or confidence) in the body of evidence for each outcome assessed. | 5-6 |
| **DISCUSSION** | | |  |
| Discussion | 23a | Provide a general interpretation of the results in the context of other evidence. | 6 |
|  | 23b | Discuss any limitations of the evidence included in the review. | 6-7 |
|  | 23c | Discuss any limitations of the review processes used. | 6-7 |
|  | 23d | Discuss implications of the results for practice, policy, and future research. | 7 |
| **OTHER INFORMATION** | | |  |
| Registration and protocol | 24a | Provide registration information for the review, including register name and registration number, or state that the review was not registered. | 3-4 |
|  | 24b | Indicate where the review protocol can be accessed, or state that a protocol was not prepared. | 3-4 |
|  | 24c | Describe and explain any amendments to information provided at registration or in the protocol. | 3-4 |
| Support | 25 | Describe sources of financial or non-financial support for the review, and the role of the funders or sponsors in the review. | 14 |
| Competing interests | 26 | Declare any competing interests of review authors. | 14 |
| Availability of data, code and other materials | 27 | Report which of the following are publicly available and where they can be found: template data collection forms; data extracted from included studies; data used for all analyses; analytic code; any other materials used in the review. | 3-4 |

# **Table S2.** Search Strategy

#1. "Spine"[Mesh] OR spine[ti] OR vertebral[ti] OR vertebra[ti] OR vertebrae[ti]

#2. "Recurrence"[Mesh] OR “fragility fracture”[tiab] OR “fragility fractures”[tiab] OR "Osteoporosis"[Mesh]

#3. #1 AND #2

#4. “randomized controlled trial”[Publication Type] OR “controlled clinical trial”[Publication Type] OR randomized[tiab] OR placebo[tiab] OR randomly[tiab] OR trial[ti] OR random*[tiab]

#5. #3 AND #4

Filters: Humans, to 2015

# **Figure S1**. Quality evaluation of randomized controlled trials


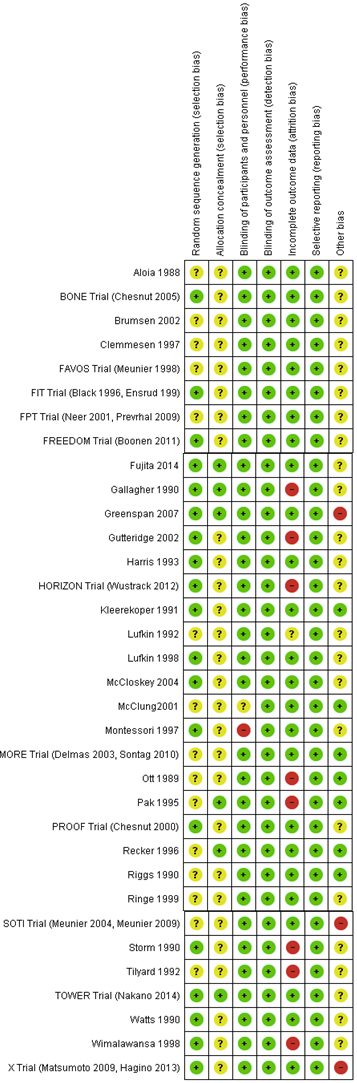

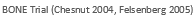


# **Figure S2.** The overall quality of randomized controlled trials


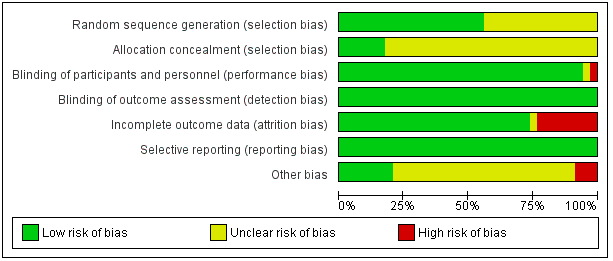


# **Figure S3.** The incidence rate of VFFs according to number of prior VFFs, expressed as number of patients every 100 person-years


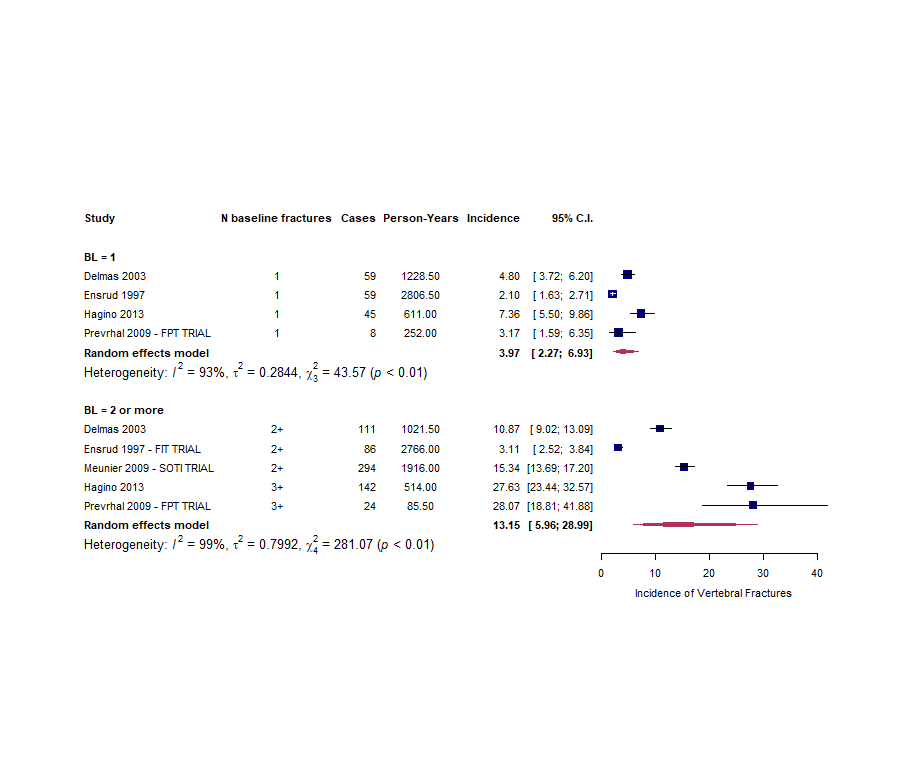
*BL=baseline*

# **
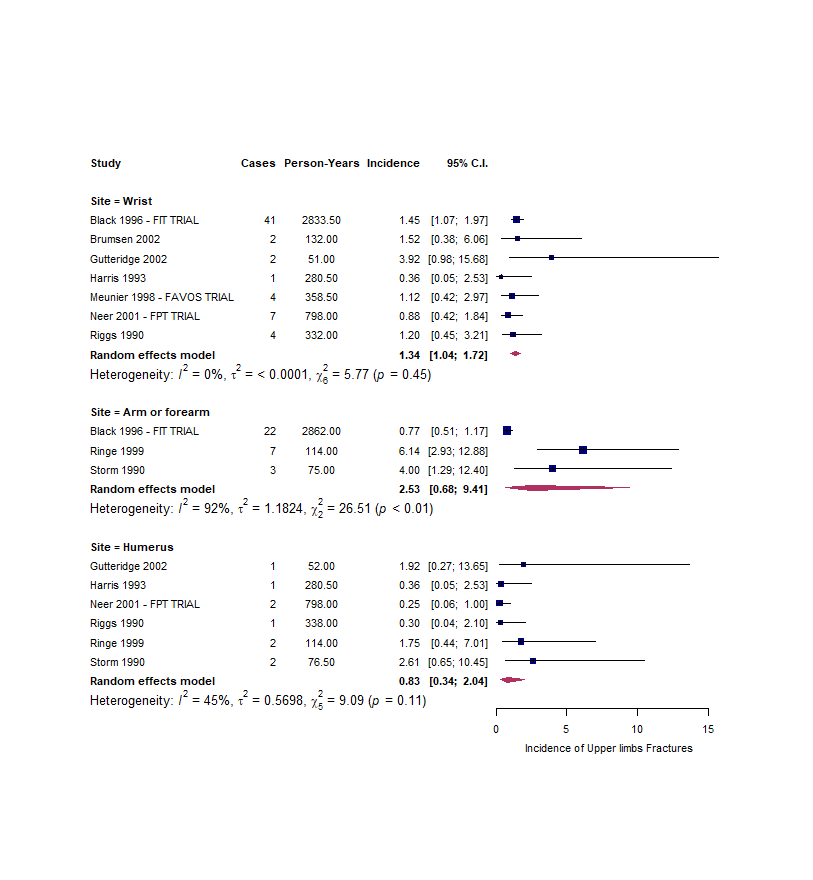
Figure S4.** The incidence rate of upper limb fractures, expressed as number of patients every 100 person-years

# **Figure S5.** The incidence rate of lower limb fractures, expressed as number of patients every 100 person-years


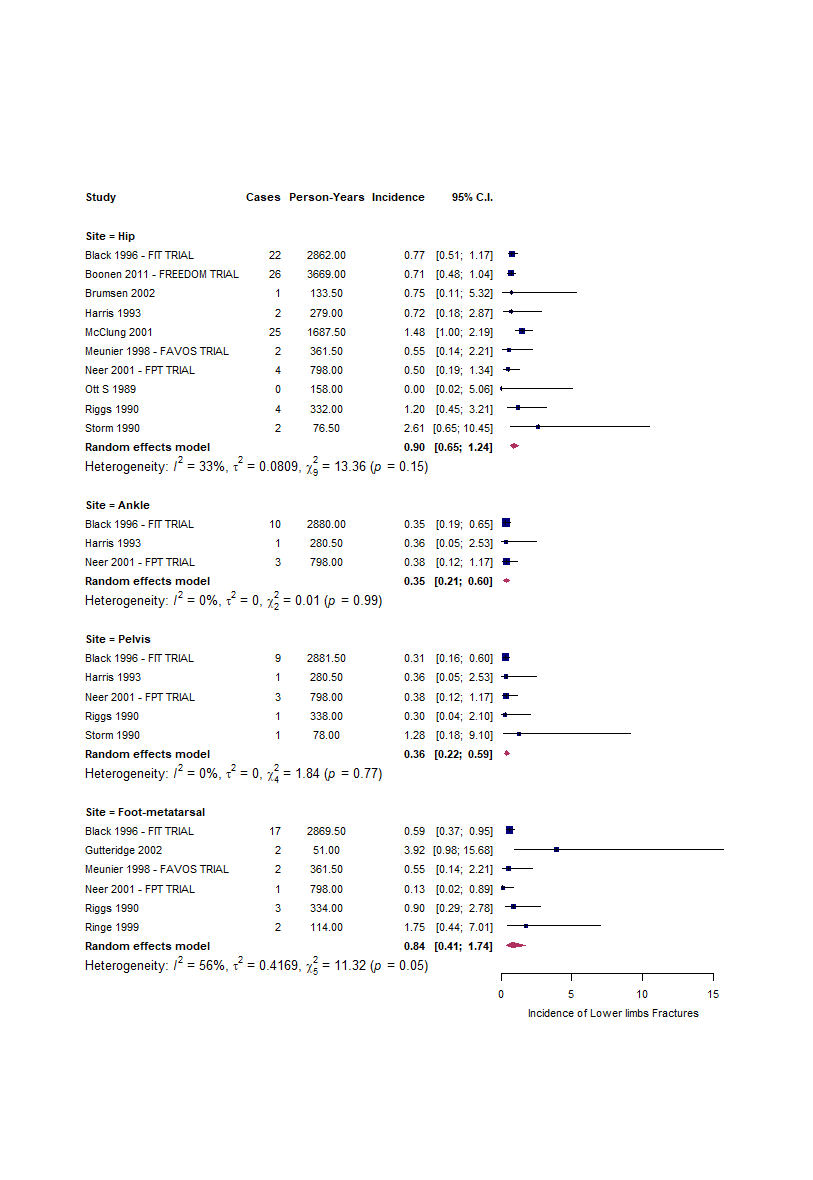


# **Figure S6.** The incidence rate of torso fractures, expressed as number of patients every 100 person-years


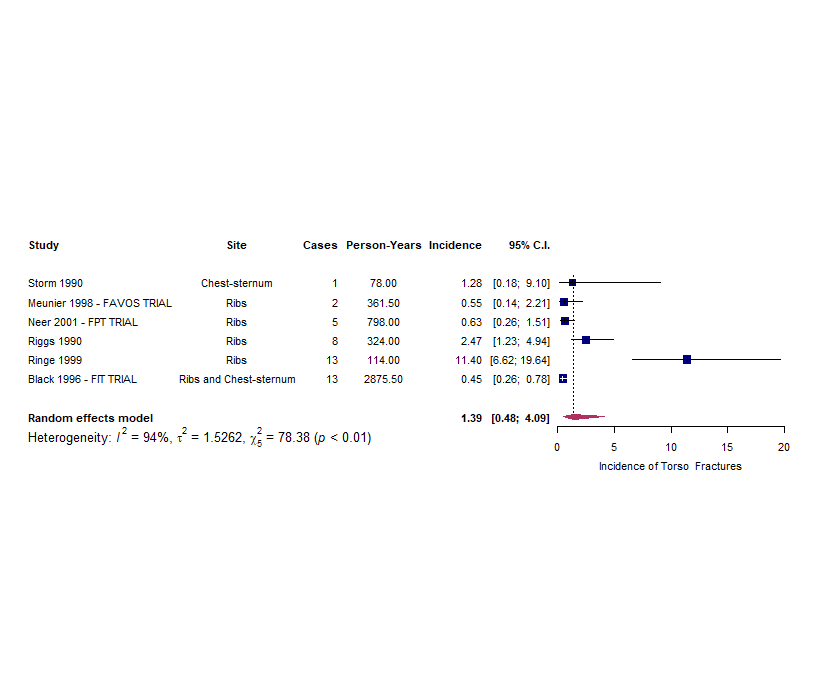


# **Figure S7.** The incidence rate of other fractures, expressed as number of patients every 100 person-years


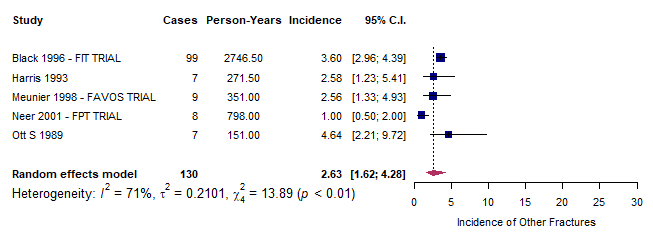


¤

¥

Ç

§

*

Ç Other than wrist, spine, or hip fracture

¤ wrist, humerus, pelvis, hip, distal femur, and ankle

¥ Other than Colles’, hip, rib, metatarsus of Calcaneus, tibia

* Other than hip, wrist, ankle, humerus, rib, foot or pelvis fracture

§ Rib, hand

# **Figure S8.** Begg’s funnel plot for publication bias in the studies assessing the incidence rate of new vertebral fracture.


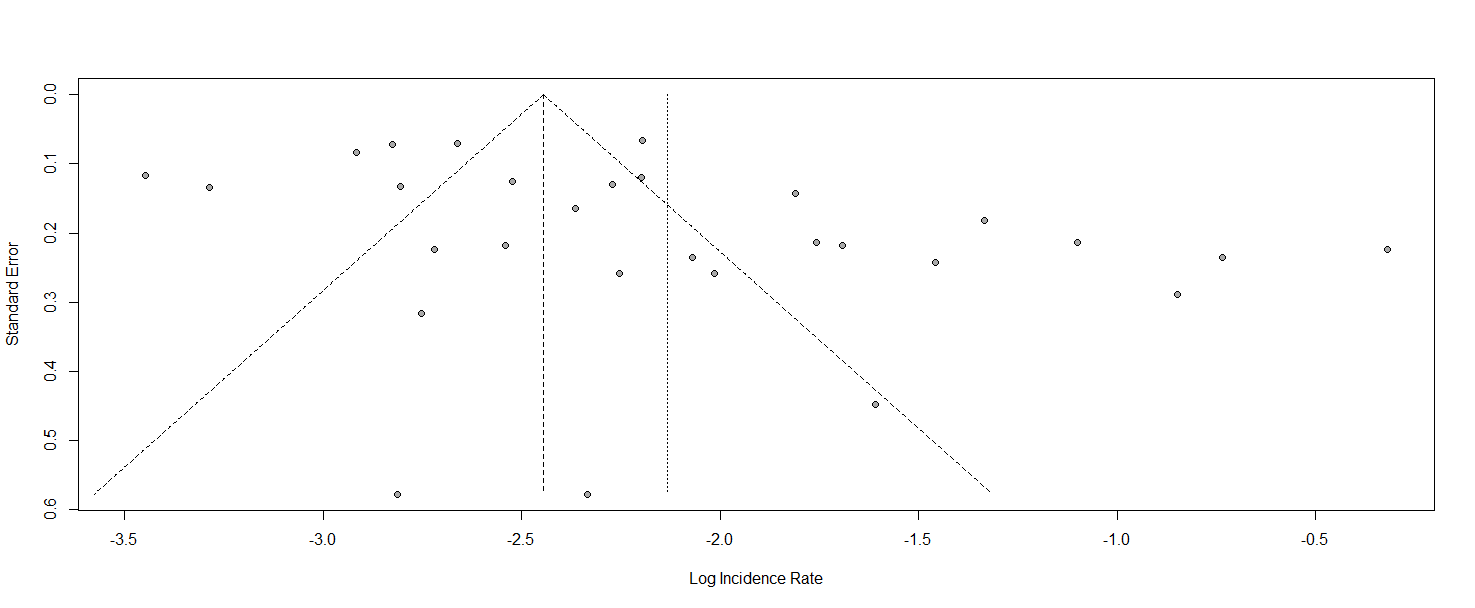


# **Figure S9.** Begg’s funnel plot for publication bias in the studies assessing the incidence rate of new non-vertebral fracture.


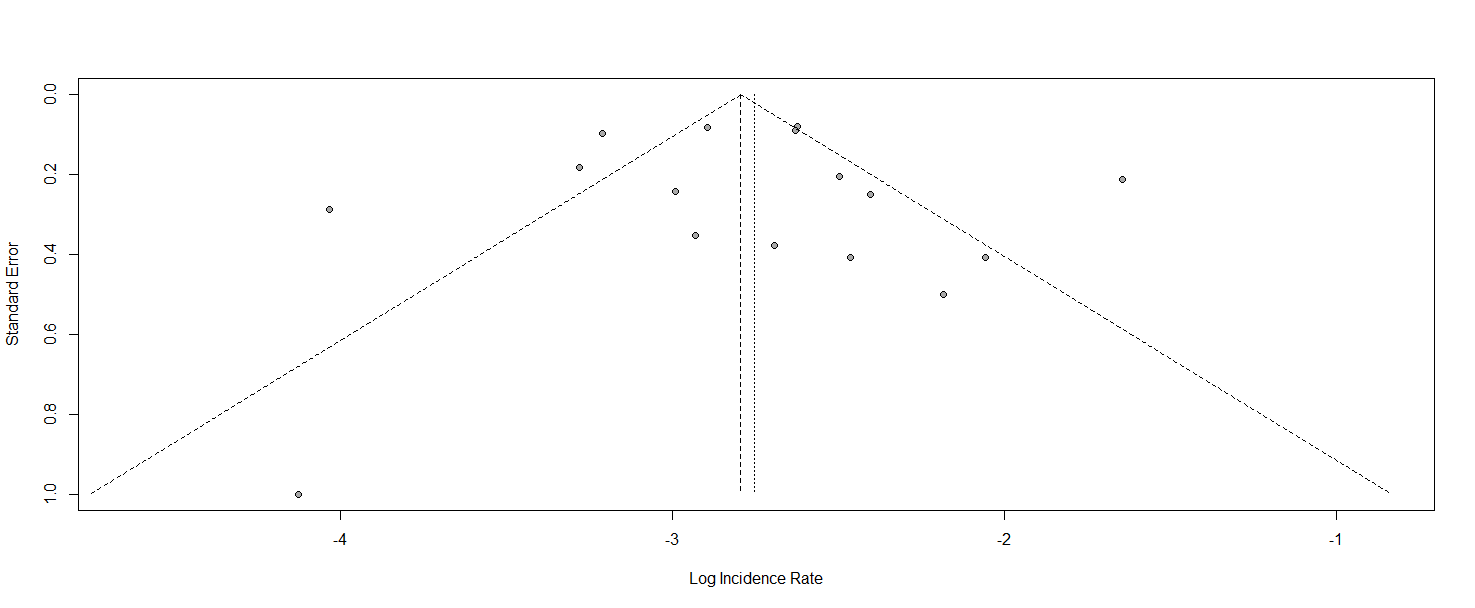


# **Figure S10.** Influence analysis investigating the pooled incidence rate of a) vertebral refracture and b) non-vertebral refracture by omitting one study at time among those contributing the estimates reported in Figure 2.

1.
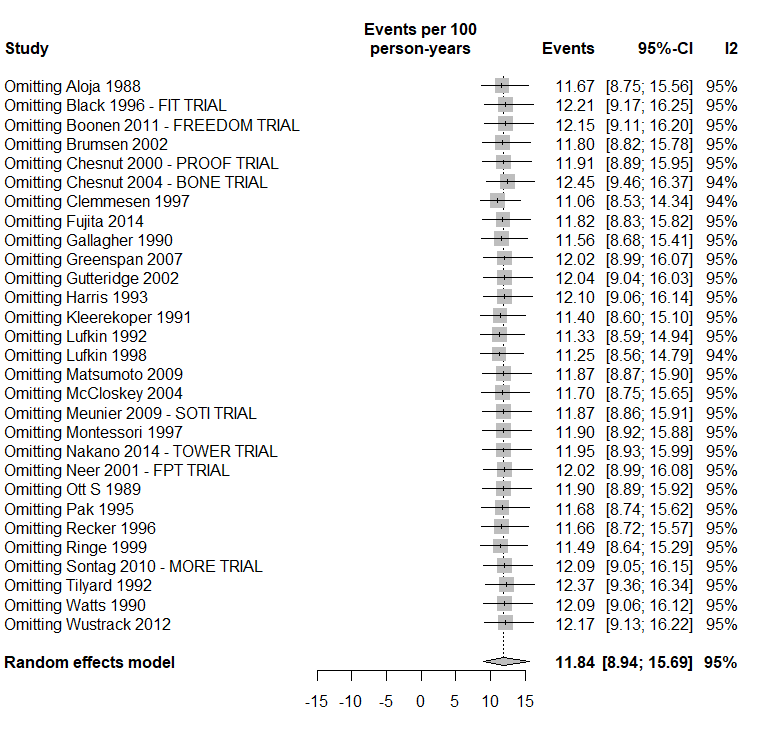
Vertebral refracture Incidence rate
2. Non-vertebral refracture Incidence rate


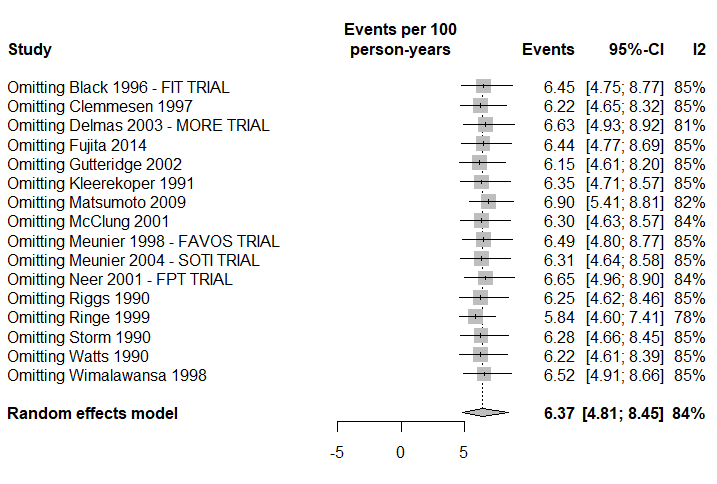


# Complete list of experts involved

**Fragility Fracture Team**

*Giovanni Adami Member of SIOMMMS - Società Italiana di Osteoporosi del metabolismo minerale e delle malattie dello scheletro*

*Rosaria Alvaro Associate Professor in Nursing Sciences – University of Rome Tor Vergata*

*Annalisa Biffi Department of Statistics and Quantitative Methods; Healthcare Research & Pharmacoepidemiology Interuniversity Center, University of Milan-Bicocca*

*Riccardo Bogini General Pratictioner at USL Umbria*

*Maria Luisa Brandi Full Professor of Endocrinology and Metabolic Bone Diseases - University of Florence; Director of the Regional Referral Center for Hereditary Endocrine Tumors; Director Clinical Unit on Metabolic Bone Disorders - University Hospital of Florence; President of FIRMO - Fondazione Italiana Ricerca sulle Malattie dell’Osso*

*Achille Patrizio Caputi Emeritus Professor of Pharmacology; University of Messina*

*Luisella Cianferotti Associate Professor of Endocrinology, University of Florence; member of FIRMO - Fondazione Italiana Ricerca sulle Malattie dell’Osso*

*Giovanni Corrao Full Professor of Medical Statistics – University of Milan-Bicocca; Director of Healthcare Research & Pharmacoepidemiology Interuniversity Center*

*Bruno Frediani Full Professor of Rheumatology; Director of the School of Specialization in Rheumatology - University of Siena; Director of the Complex Operational Unit in Rheumatology; Director of the Department of Medical Sciences*

*Davide Gatti Associate Professor of Rheumatology – University of Verona; President of the Scientific Committee of the ASITOI - Associazione Italiana Osteogenesi Imperfetta; Coordinator of the Guideline Commission SIOMMMS - Società Italiana di Osteoporosi del metabolismo minerale e delle malattie dello scheletro*

*Stefano Gonnelli Full Professor of Internal Medicine and Director of the School of Specialization in Iternal Medicine - University of Siena*

*Giovanni Iolascon Full Professor of Physical and Rehabilitation Medicine – University of Campania “Luigi Vanvitelli”*

*Andrea Lenzi Full Professor of Endocrinology - University of Rome La Sapienza; member of SIE – Società italiana di Endocrinologia*

*Salvatore Leone Member of AMICI Onlus - Associazione nazionale per le Malattie Infiammatorie Croniche dell'Intestino*

*Raffaella Michieli National Secretary SIMG – Società italiana di medicina generale e delle cure primarie*

*Silvia Migliaccio Member of SIE – Società italiana di Endocrinologia; Associate Professor - University of Rome Foro Italico*

*Tiziana Nicoletti Manager of CnAMC - Coordinamento nazionale delle Associazioni dei Malati Cronici e rari di Cittadinanzattiva*

*Marco Paoletta Member of SIMFER - Società Italiana di Medicina Fisica e Riabilitativa*

*Annalisa Pennini Member of FNOPI - Federazione Nazionale degli Ordini delle Professioni Infermieristiche per il progetto Fratture da Fragilità*

*Eleonora Piccirilli Department of Orthopedics and Traumatology, University of Rome Tor Vergata*

*Gloria Porcu Department of Statistics and Quantitative Methods; Healthcare Research & Pharmacoepidemiology Interuniversity Center, University of Milan-Bicocca; Unit of Biostatistics, Epidemiology and Public Health, Department of Cardiac, Thoracic, Vascular Sciences and Public Health, University of Padua, Padua, Italy*

*Raffaella Ronco Department of Statistics and Quantitative Methods; Healthcare Research & Pharmacoepidemiology Interuniversity Center, University of Milan-Bicocca*

*Maurizio Rossini Full Professor of Rheumatology; President of SIOMMMS - Società Italiana dell'Osteoporosi, del Metabolismo Minerale e delle Malattie dello Scheletro; Member of SIR - Società Italiana di Reumatologia*

*Umberto Tarantino Full Professor of Diseases of the Locomotor System – University of Rome; Member of SIOT – Società italiana di ortopedia e traumatologia*
